# Targeted therapeutic effect against the breast cancer cell line MCF-7 with a CuFe_2_O_4_/silica/cisplatin nanocomposite formulation

**DOI:** 10.3762/bjnano.10.214

**Published:** 2019-11-12

**Authors:** B Rabindran Jermy, Vijaya Ravinayagam, Widyan A Alamoudi, Dana Almohazey, Hatim Dafalla, Lina Hussain Allehaibi, Abdulhadi Baykal, Muhammet S Toprak, Thirunavukkarasu Somanathan

**Affiliations:** 1 Department of Nano-Medicine Research, Institute for Research and Medical Consultations, Imam Abdulrahman Bin Faisal University, P.O. Box 1982, 31441 Dammam, Saudi Arabia; 2 Deanship of Scientific Research & Department of Nano-Medicine Research, Institute for Research and Medical Consultations, Imam Abdulrahman Bin Faisal University, P.O. Box 1982, 31441 Dammam, Saudi Arabia; 3 Department of Neuroscience Research, Institute for Research and Medical Consultations, Imam Abdulrahman Bin Faisal University, P.O. Box 1982, 31441 Dammam, Saudi Arabia; 4 Department of Stem Cell Research, Institute for Research and Medical Consultations, Imam Abdulrahman Bin Faisal University, P.O. Box 1982, 31441 Dammam, Saudi Arabia; 5 College of Engineering Research (CER), King Fahd University of Petroleum and Minerals, 31261 Dhahran, Saudi Arabia; 6 PharmD, College of Clinical Pharmacy, Imam Abdulrahman Bin Faisal University, Dammam, Saudi Arabia; 7 Department of Materials and Nano Physics, KTH-Royal Institute of Technology, 16440 Stockholm, Sweden; 8 Department of Chemistry, School of Basic Sciences, Vels Institute of Science, Technology and Advanced Studies (VISTAS), Chennai 600117, India

**Keywords:** anticancer, cisplatin, copper ferrite, drug delivery, multifunctional, nanomedicine, nanotherapeutics, spherical silica, tumour therapy

## Abstract

The combination of magnetic nanoparticles with a porous silica is a composite that has attracted significant attention for potential multifunctional theranostic applications. In this study, 30 wt % CuFe_2_O_4_ was impregnated into a matrix of monodispersed spherical hydrophilic silica (HYPS) nanoparticles through a simple dry impregnation technique. The chemotherapy drug cisplatin was loaded through electrostatic equilibrium adsorption over 24 h in normal saline solution. The presence of cubic spinel CuFe_2_O_4_ on HYPS was confirmed through powder X-ray diffraction (PXRD), Fourier transform infrared spectroscopy (FTIR) and diffuse reflectance UV–vis spectroscopy (DR UV–vis) analysis. The HYPS particles showed a surface area of 170 m^2^/g, pore size of 8.3 nm and pore volume of 0.35 cm^3^/g. The cisplatin/CuFe_2_O_4_/HYPS nanoformulation showed the accumulation of copper ferrite nanoparticles on the surface and in the pores of HYPS with a surface area of 45 m^2^/g, pore size of 16 nm and pore volume of 0.18 cm^3^/g. Transmission electron microscopy (TEM) and energy dispersive X-ray (EDX) mapping analysis showed the presence of homogeneous silica particles with nanoclusters of copper ferrite distributed on the HYPS support. Vibrating sample magnetometry (VSM) analysis of CuFe_2_O_4_/HYPS showed paramagnetic behavior with a saturated magnetization value of 7.65 emu/g. DRS UV–vis analysis revealed the functionalization of cisplatin in tetrahedral and octahedral coordination in the CuFe_2_O_4_/HYPS composite. Compared to other supports such as mesocellular foam and silicalite, the release of cisplatin using the dialysis membrane technique was found to be superior when CuFe_2_O_4_/HYPS was applied as the support. An in vitro experiment was conducted to determine the potential of CuFe_2_O_4_/HYPS as an anticancer agent against the human breast cancer cell line MCF-7. The results show that the nanoparticle formulation can effectively target cancerous cells and could be an effective tumor imaging guide and drug delivery system.

## Introduction

Due to the continuous advancements in the field of nanotechnology, the therapeutic prospects have been broadened in terms of chronic cancer, diabetic and other metabolic disorders. The global burden of cancer is expected to affect about 24 million people by 2035. The robust technological advancements in utilizing nanoparticles for therapeutic applications are the new hope to circumvent the expected health crisis [1]. However, the single modal drug delivery system is hampered by low bioavailability (about 5–10%), burst release, and lower target efficiency. Multifunctional theranostic nanoparticles that can respond to an external magnetic field for drug release and assist in bioimaging (magnetic resonance imaging), tissue repair, and thermal ablation have been gaining considerable attention in recent years. In particular, the use of superparamagnetic iron oxide nanoparticles (SPIONs) is now advantageous as they are FDA-approved for clinical use [2]. Magnetic Fe_3_O_4_-based mesoporous silica materials have been reported to be effective for cancer therapeutics [3–4]. However, due to the poor crystallinity of SPIONs on silica supports, a low saturation value of magnetization occurs in the silica bound nanocomposites. For instance, the magnetometer (superconducting quantum interference device (SQUID)) analysis of silica/iron oxide nanocomposites showed the magnetization of 1.65 emu/g. Recently, we have showed that micrometer-sized spherical silica exhibit the highest magnetization of 1.44 emu/g, while silicalite showed the lowest value of 0.08 emu/g, respectively [5]. Although the saturated magnetization can be increased with a high loading of SPIONs, the formation of a mixture of iron oxide species (α-Fe_2_O_4_, Fe_3_O_4_ and γ-Fe_2_O_4_) becomes inevitable.

However, the surface of iron oxide can be modified with various transition heteroatoms including Ni, Mn, Co, and Cu, leading to family of spinel ferrites. The resulting magnetic nanoferrites are inexpensive and can be easily prepared without multistep protocols. Ferromagnetism was reported to occur due to antiparallel spins of Fe^3+^ located at tetrahedral sites, while M^2+^ are located at octahedral sites. Recently, we have reported the low-cost preparation of a copper nanoferrite using a citrate sol–gel technique [6]. In addition, several metal-based ferrite systems have been recently reported for bioscience applications [7]. Although spinel ferrite nanoparticles are interesting, due to the strong magnetic dipole–dipole interactions, they tend to form aggregates, exert variable oxidation states and result in dose-dependent toxicity. Several supports, including silica and carbon, were used to reduce such aggregation. Notably, different types of nanocomposites were reported based on Co, Ni, Mn and Fe over mesoporous carbon capsules [8]. A carbon-shell support thickness of about 50 nm was reported with a high ferrite loading capacity of about 30–50 wt % with particle diameters between 9 and 17 nm (at the external carbon layer). Such a magnetic nanoformulation was found to be useful for enzyme lysozyme immobilization. The magnetic properties of ZnFe_2_O_4_/MCM-41 and NiFe_2_O_4_/MCM-41 synthesized via the wet impregnation technique has been studied and compared with bare ferrites. The findings show that ferrites enclosed inside the non-magnetic hexagonal pores of MCM-41 exhibit less dipolar interactions, which reduces the magnetization value due to the surface anisotropy effect [9–10]. Among the ferrites, copper ferrites are interesting due to their superior conductive and magnetic characteristics. A mesoporous Cu_1−_*_x_*Zn*_x_*Fe_2_O_4_ system has been reported using a nanocasting technique [11]. Based on the Bertaut analysis, the doping of Zn tends to form mixed inverse spinels occupying the A site, while Cu^2+^ prefers the B site. SQUID-vibrating sample magnetometry (VSM) analysis showed the formation of superparamagnetic behavior, while a temperature dependence study using field cooling and zero field cooling analysis (ZFC/FC) showed spin-glass-like surface layers in the mixed metal oxide spinel composite. CuFe_2_O_4_ composite formation with activated carbon was reported using the co-precipitation technique [12–13].

**Scheme 1:**
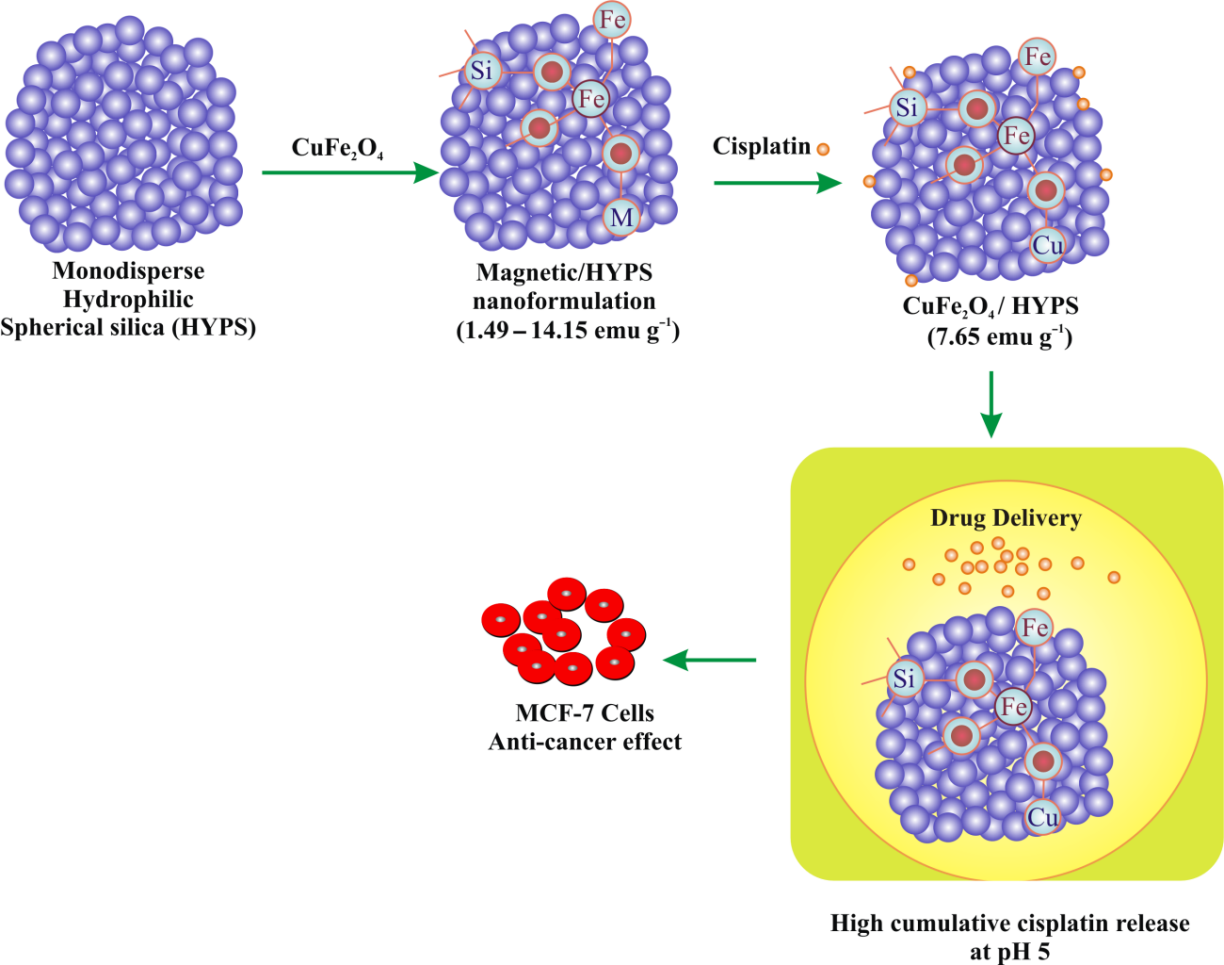
Schematic representation of CuFe_2_O_4_/HYPS/cisplatin nanoformulation.

Herein, we have prepared a nanocomposite comprised of CuFe_2_O_4_ and monodispersed spherical hydrophilic silica (HYPS) particles (Scheme 1) for multifunctional biomedical applications. The crystalline phase, morphology, magnetization, and coordination environment of various spinel species were characterized using X-ray diffraction (XRD), BET surface area measurements, vibrating sample magnetometry (VSM), diffuse reflectance UV–vis spectroscopy (DR UV–vis), scanning electron microscopy (SEM) equipped with energy dispersive X-ray (EDX) spectroscopy and transmission electron microscopy (TEM) techniques. The study showed the high cisplatin release capability and targeted anticancer efficiency demonstrated in vitro in the breast cancer cell line MCF-7.

## Materials and Methods

HYPS was purchased from Superior Silica, USA. Cu(NO_3_)_2_·3H_2_O, Fe(NO_3_)_3_·9H_2_O, cisplatin and aluminium mesocellular foam with a high surface area of 539 m^2^/g and large aperture pore size of 14.7 nm was obtained from Sigma-Aldrich. Silicalite with a surface area of 313 m^2^/g was prepared in-house using tetraethyl orthosilicalite and tetrapropylammonium hydroxide as the silica source and template. All chemicals were used as-receied without any further modification or purification.

### Preparation of 30 wt % CuFe_2_O_4_/HYPS

The sample was prepared by mixing 0.65 g of Cu(NO_3_)_2_·3H_2_O and 1.01 g of Fe(NO_3_)_3_·9H_2_O with 1.4 g of predried support material (HYPS, AlMSU-F and silicalite) using a mortar and pestle for 30 min followed by calcination at 850 °C for 6 h. Cisplatin was functionalized by mixing 30 mg of cisplatin with 600 mg of CuFe_2_O_4_/HYPS in normal saline solution (NSS) in an ice-cooled dark environment. After stirring overnight, the solution mixture was filtered and washed with 15 mL of normal saline solution. Then the adsorbed cisplatin was calculated using UV–visible spectroscopy at 208 nm [14]. Depending on the support, the loaded formulations were labelled as CuFe_2_O_4_/HYPS, CuFe_2_O_4_/AlMSU-F and CuFe_2_O_4_/silicalite.

### Characterization techniques

Powder X-ray diffraction (PXRD) patterns for the CuFe_2_O_4_/HYPS nanoformulation were analyzed using a Miniflex 600 instrument (Rigaku, Japan). The surface textures the formulations were analyzed with a ASAP-2020 plus (Micromeritics, USA) instrument. Copper ferrite and cisplatin functional groups were identified using FTIR using ATR technology (Perkin Elmer, USA). The morphological features of the nanoformulations were identified by SEM and TEM. The elemental distribution in the samples was investigated using SEM-EDS. SEM was performed using a JSM-6610LV instrument from JEOL. The prepared powder was dispersed onto a doubled sided tape holder and examined under 20 keV. EDS spectra were obtained using Aztec software from Oxford company. The suspensions for TEM analysis were prepared from the dry sample using ethanol followed by ultrasonic treatment for 30 min. A droplet (5 µL) of diluted suspension was deposited in a 300-mesh pure carbon grid and then kept in a pumping station for 1 h for further drying. The grids were examined by the JEM2100F instrument from JEOL.

### Drug release study

The cumulative cisplatin release was studied using CuFe_2_O_4_/HYPS, CuFe_2_O_4_/AlMSU-F and CuFe_2_O_4_/silicalite nanoformulations. The cellulose membrane dialysis tubing was activated, and drug delivery was performed by immersing the bag containing 30 mg of drug formulations in 50 mL of phosphate buffered saline (PBS) at pH 5.6. The release was performed under constant temperature at 37 °C. At regular time intervals, a specific volume of solution was removed (10 mL) and analyzed using UV–vis spectroscopy. The withdrawn solution was replaced with an equal volume of fresh PBS solution.

### In vitro study on MCF-7 cells

In this study, the antitumor effect of cisplatin-loaded 30 wt % CuFe_2_O_4_/HYPS material was tested on the human mammary adenocarcinoma cell line, MCF-7. The cells were maintained in Dulbecco’s Modified Eagle Medium (DMEM) (Gibco, Life Technologies) supplemented with 10% heat-inactivated fetal bovine serum (HI-FBS) (Gibco, Life Technologies), 1% penicillin streptomycin (100× Gibco, Life Technologies), and 1% MEM non-essential amino acids (NEAA) (100× Gibco, Life Technologies). The cells were kept in a humidified incubator at 37 °C with 5% CO_2_. For the experimental setup, MCF-7 cells were seeded in a 96-well plate at a density of 20,000 cells/well. On the next day, the cells were changed to the starve media (0.5% HI-FBS containing media) for 24 h before treatment. The cells were then treated for 48 h with the following: CuFe_2_O_4_, HYPS, CuFe_2_O_4_/HYPS, cisplatin, and cisplatin/CuFe_2_O_4_/HYPS. For the HYPS, CuFe_2_O_4_/HYPS, and cisplatin/CuFe_2_O_4_/HYPS groups, the treatment concentrations were as follows: 0.025, 0.05, 0.1, and 0.5 mg/mL. The concentration of free CuFe_2_O_4_ and free cisplatin was calculated to reflect the amount that would have been adsorbed on the resulting cisplatin/CuFe_2_O_4_/HYPS nanoparticles. Based on the drug loading experiments, there was 0.3357 mg of CuFe_2_O_4_ and 0.045 mg of cisplatin in 1 mg of HYPS. Therefore, if the experimental concentration of cisplatin/CuFe_2_O_4_/HYPS was 0.025 mg/mL, then the actual concentration of adsorbed CuFe_2_O_4_ on these nanoparticles is 0.0084 mg/mL. Similarly, the adsorbed cisplatin on these nanoparticles is 0.001125 mg/mL. The same calculation was made for the other doses as indicated in Table 1. Thus, the treatment concentrations used in this experiment for CuFe_2_O_4_ were: 0.0084, 0.0168, 0.0336, and 0.168 mg/mL. The treatment concentrations for cisplatin were: 0.001125, 0.00225, 0.0045, 0.0225 mg/mL.

**Table 1:** Experimental concentrations for in vitro study.

Concentration of HYPS (mg/mL)	Corresponding concentration of adsorbed CuFe_2_O_4_ (mg/mL)	Corresponding concentration of adsorbed cisplatin (mg/mL)

0.025	0.0084	0.001125
0.05	0.0168	0.00225
0.1	0.0336	0.0045
0.5	0.168	0.0225

### Cell viability – MTT assay

The cell viability was tested using 3-(4,5-dimethylthiazol-2-yl)-2,5-diphenyltetrazolium bromide (MTT) assay, which is based on the ability to reduce MTT to formazan crystals. The assay was performed using previously published protocols [15]. Briefly, MTT (Sigma-Aldrich) was dissolved in PBS at 5 mg/mL. The working solution of MTT was prepared at a final concentration of 0.5 mg/mL (10 µL of stock MTT + 90 µL 1× PBS/well). The 96 well plate was washed twice with 1× PBS and 100 µL of the MTT working solution was dispensed in all wells. An MTT background control was included in which the MTT working solution was added to empty wells (no cells). The plate was incubated for three hours at 37 °C, followed by the addition of 100 µL of acidified isopropanol solubilizing solution (0.04 N HCL isopropanol). The change in color intensity was measured at 570 nm using a SYNERGY-neo2 BioTek ELISA reader. Each condition was performed in triplicate and the reading of each triplicate was averaged and subtracted from the averaged MTT background control reading. Each condition was compared to the control (no treatment) wells. The following equation was used to calculate the percent of cell viability:


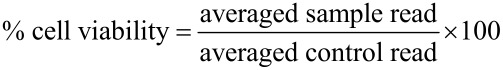


### Statistical analysis

The cell viability assay data contain four independent experiments. The statistical analysis was performed using Prism 7 software (GraphPad, La Jolla, CA). The analysis was performed using two-way ANOVA with Dunnett’s post hoc test. Error bars ± standard error of the mean (SEM) and * *p* < 0.05; ** *p* < 0.01; *** *p* < 0.001; **** *p* < 0.0001 versus control. N.S. indicates non-significant.

## Results and Discussion

**Figure 1:**
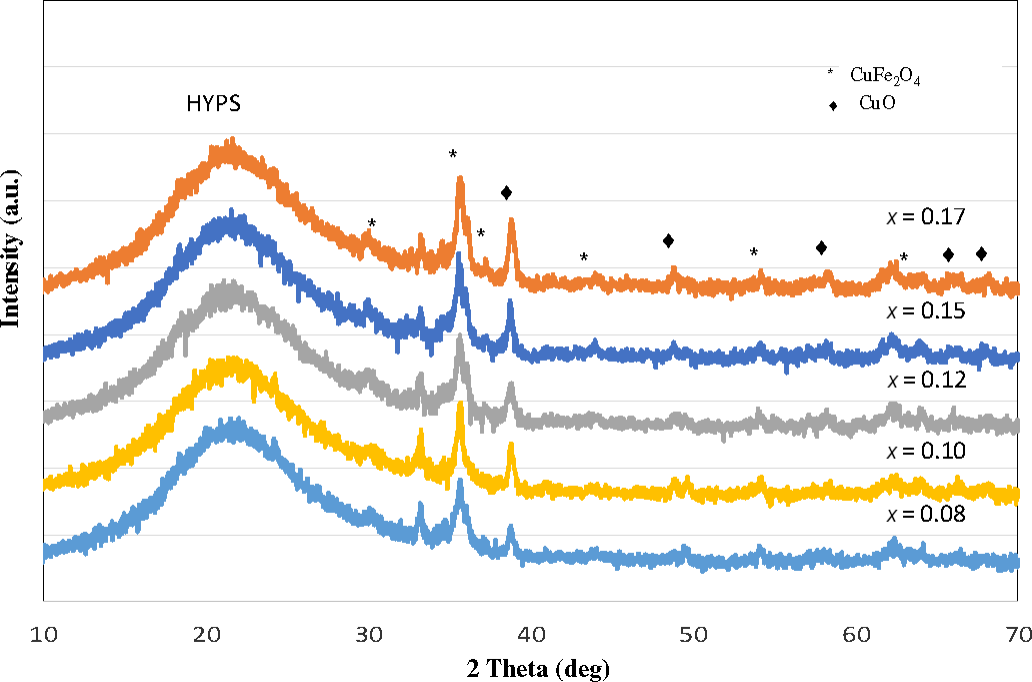
Powder XRD patterns of CuFe_2_O_4_/HYPS with different Cu concentrations (*x* = 0.08, 0.10, 0.12, 0.15 and 0.17).

Figure 1 shows the PXRD patterns of 30 wt % CuFe_2_O_4_ loaded onto HYPS using the dry impregnation technique. The presence of broad peaks due to the amorphous nature of the siliceous framework of HYPS was observed between 15–30°. For the metal oxides, the diffraction patterns correlate with the cubic phase of copper ferrite (JCPDS 77-0010) and CuO. The presence of broad CuFe_2_O_4_ peaks demonstrates the formation of nanometer-sized particles at the nanopores of HYPS. In order to optimize the saturation magnetization value, the copper content (*x* = 0.08–0.17) was varied over HYPS (Table 2). The intensity of the copper cubic ferrite peak was found to increase with the copper content over HYPS with an increase of *x* from 0.08 to 0.17. This shows the importance of occupation of copper in the octahedral position to improve the crystallinity. Such stabilization of the cubic phase of CuFe_2_O_4_ inside the pore channels of silica have been reported due to suppression of John–Teller distortion [16].

**Table 2:** The stoichiometric amount of CuFe_2_O_4_/silica nanocomposite.

Silica support	Cu (g)	Fe (g)	Silica (g)

HYPS	0.17	0.14	1.4
HYPS	0.15	0.14	1.4
HYPS	0.12	0.14	1.4
HYPS	0.10	0.14	1.4
HYPS	0.08	0.14	1.4
AlMSU-F	0.17	0.14	1.4
silicalite	0.17	0.14	1.4

**Figure 2:**
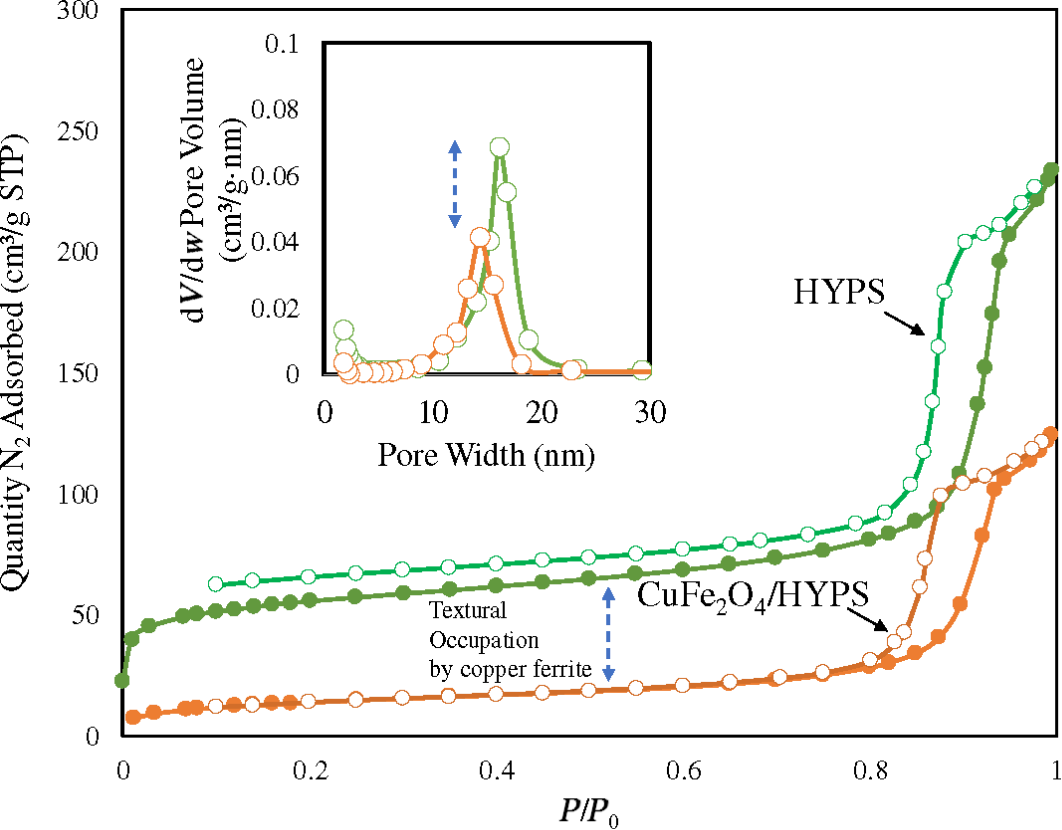
BET adsorption–desorption isotherm and pore size distribution of (a) HYPS and (b) 30 wt% CuFe_2_O_4_/HYPS.

The surface area and pore size distribution of HYPS and CuFe_2_O_4_/HYPS were analyzed using BET theory and the nitrogen adsorption technique (Figure 2). HYPS particles show a type IV isotherm corresponding to the presence of mesopores. The silica hysteresis loop tends to be present at a higher relative pressure of *P*/*P*_0_ > 0.8. The HYPS texture exhibited a surface area of 170 m^2^/g, pore volume of 0.35 cm^3^/g with an intermediate average pore size of 8.3 nm. After spinel loading, the surface area decreased to 47 m^2^/g, indicating about 28% reduction in the BET surface area. In terms of pore volume occupation, a significant decrease occurs from 0.35 to 0.18 cm^3^/g. The trend clearly shows the accumulation of copper ferrite nanoparticles at the pores of HYPS.

**Figure 3:**
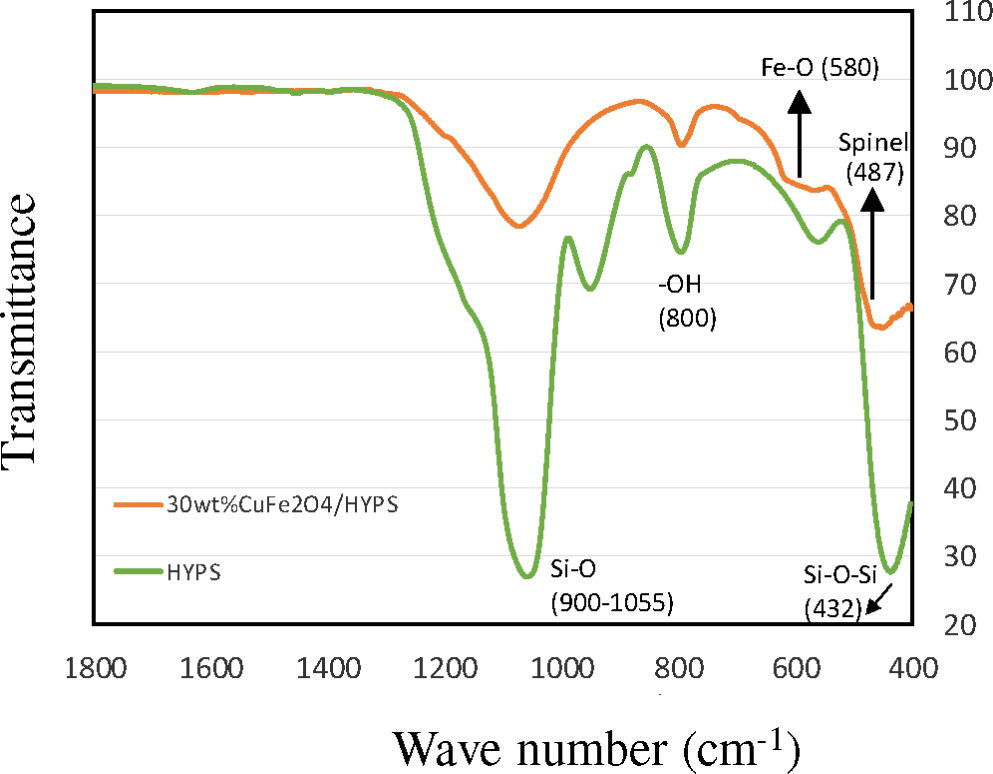
FTIR spectra of HYPS and 30 wt % CuFe_2_O_4_/HYPS.

The FTIR technique was used to confirm the bonding and vibrational modes of copper ferrite with silica (Figure 3). The FTIR spectra of HYPS showed several peaks corresponding to Si–O–Si stretching and vibration, hydroxyl and Si–O bonding around 432 cm^−1^, 800 cm^−1^ and between 900–1055 cm^−1^, respectively. Notably, the intense peak of silica around 432 cm^−1^ and 900–1055 cm^−1^ was due to silanol groups and found to decrease after CuFe_2_O_4_ deposition. Such a pattern clearly indicates the external surface occupation of spinel over HYPS. The peak position of CuFe_2_O_4_/HYPS shows the rearrangement of the peak position at about 487 cm^−1^ with the tetrahedral and octahedral sites of MFe_2_O_4_. The absorption band at 580 cm^−1^ clearly shows the presence of an Fe–O bond of spinel present over the HYPS support (Figure 3).

**Figure 4:**
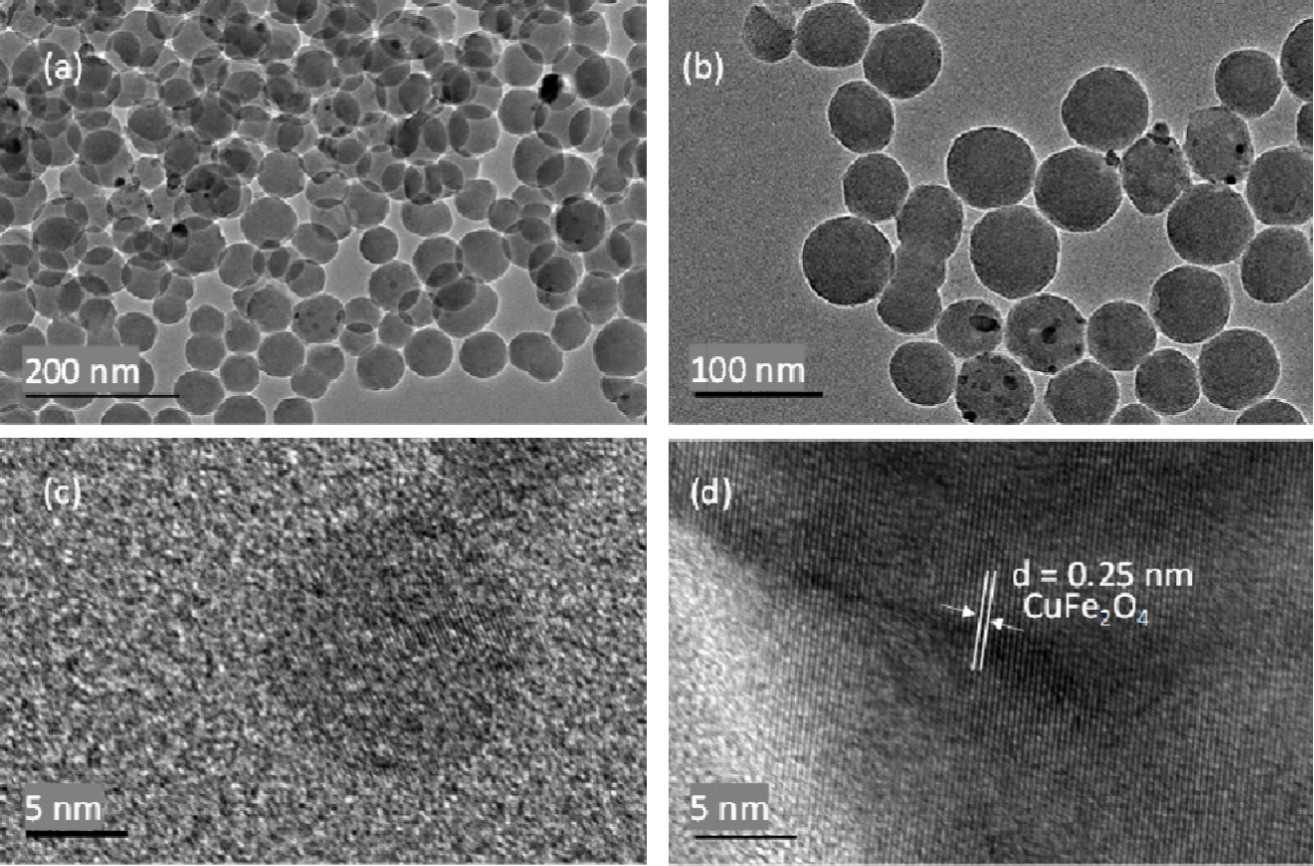
Transmission electron microscopy of (a, b) 30 wt % CuFe_2_O_4_/HYPS at different scale magnifications and (c, d) high-resolution TEM (HRTEM) images of CuFe_2_O_4_/HYPS.

The surface morphology of CuFe_2_O_4_/HYPS was analyzed using TEM and SEM-EDX (Figure 4 and Supporting Information File 1, Figure S1). TEM images of HYPS at different magnifications showed the presence of monodispersed spherical silica distributed uniformly in the range of about 80 nm (Figure 4a and 4b). The presence of spinel as agglomerated nanoclusters was clearly observed over the spherical silica. High-resolution TEM images display the lattice fringes characteristic of nanometer-sized CuFe_2_O_4_ over HYPS. The nanometer-sized parent spherical spheres were found to be interrelated with nanospinels ranging between 10 and 15 nm (Figure 4c and 4d). The measured interplanar distance was 0.25 nm, which is typical for the 311-plane of tetragonal copper ferrite. The CuFe_2_O_4_ nanoparticles were microscopically captured using EDX-mapping analysis to observe the location and nature of Cu and iron oxide dispersion over the HYPS support. The mapping showed homogeneous silica particles consistent with the TEM analysis. For the copper ferrite nanoparticles, a homogeneous mixed metal oxide formation occurs as a major proportion coexisting with Cu nanoclusters (Supporting Information File 1, Figure S1).

**Figure 5:**
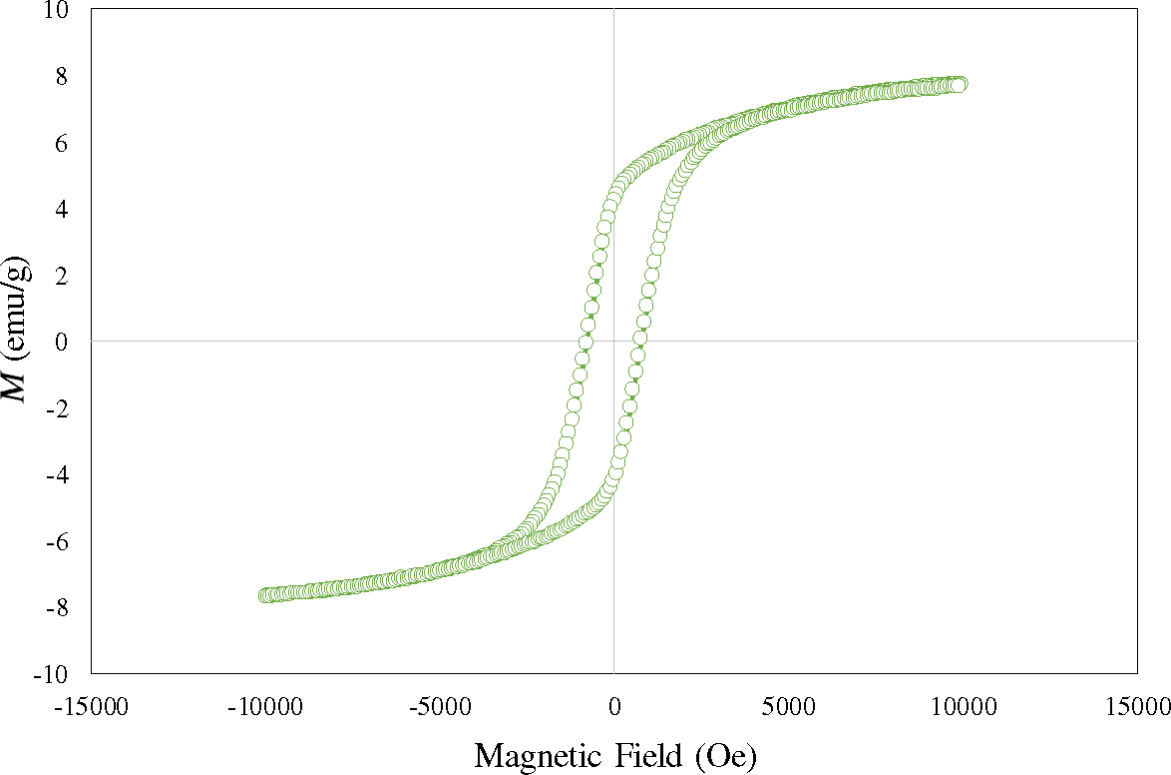
Vibrating sample magnetometer spectrum of 30 wt % CuFe_2_O_4_/HYPS.

The magnetic properties of CuFe_2_O_4_/HYPS nanocomposites were measured using VSM (Figure 5). The spinel properties are influenced by the cation distribution over the A and B sites, where the presence of different cations tends to influence the magnetic and electrical properties. Copper ferrites are well known ferrites, where Zn can be substituted at the tetrahedral site of Cu, in order to induce variable magnetization properties. The saturation magnetization value for the corresponding CuFe_2_O_4_/HYPS samples was analyzed using VSM. The sample showed paramagnetic behavior, which was reported to occur due to antiparallel spins of Fe^3+^ located at tetrahedral sites. It has been shown that the presence of small-sized nanoclusters at the walls of hexagonal-shaped MCM-41 tends to form superparamagnetic interactions among Fe^3+^ species, while large nanoclusters contribute to ferromagnetic properties [17]. In our study, paramagnetic behavior with narrow hysteresis demonstrates the formation of small nanometer-sized copper spinel clusters over HYPS (Figure 5). The particle size reduction tends to decrease the saturation magnetization. In particular, noncollinear spin arrangements present at the external nanoparticle surface play a major role, which in influences the response of the material to the external magnetic field [18]. In the case of CuFe_2_O_4_ with *x* value between 0.08 and 0.15, a lower saturated magnetization value was observed (≤1.0 emu/g), while increasing the *x* value to 0.17 showed a high magnetization of 7.65 emu/g.

**Figure 6:**
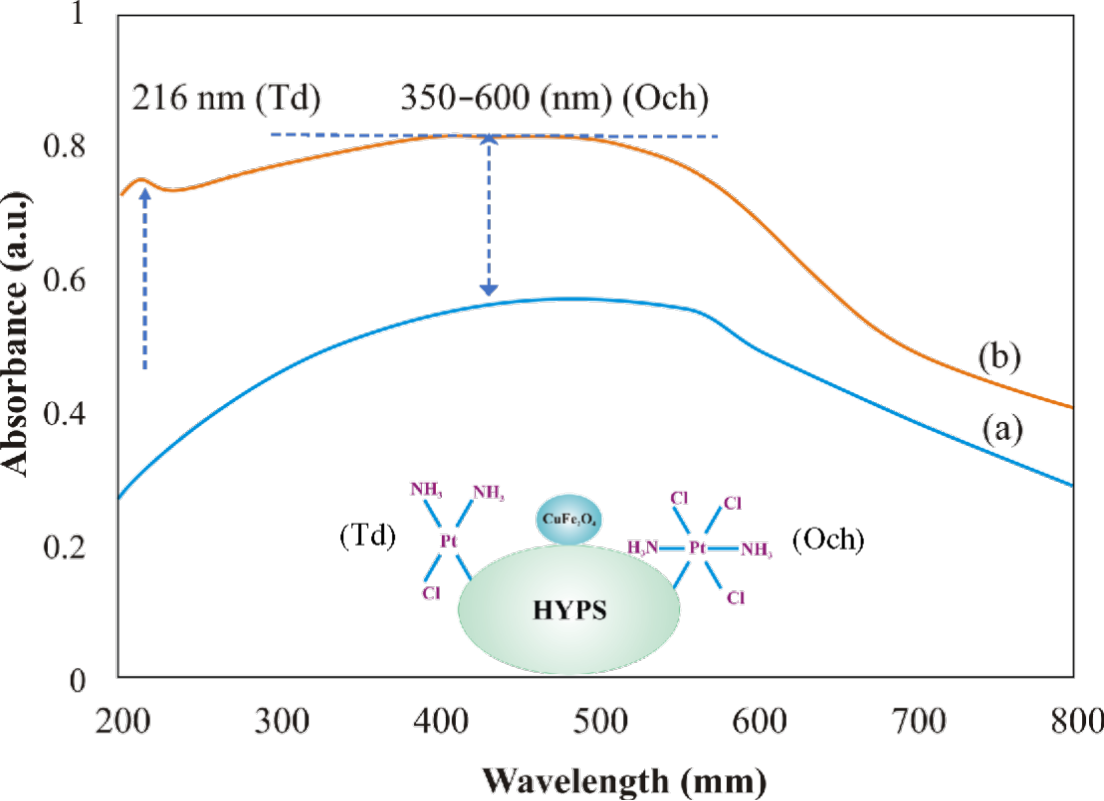
DR-UV–visible spectra of (a) CuFe_2_O_4_/HYPS and (b) cisplatin/CuFe_2_O_4_/HYPS.

In order to understand the cisplatin coordination environment of CuFe_2_O_4_/HYPS, the sample was analyzed using diffuse reflectance spectroscopy. Ferrite is a cubic spinel consisting of tetrahedral and octahedral crystalline sites [19]. Before cisplatin functionalization, a wide and strong absorption was observed between 200 and 700 nm that is characteristic of the spinel structure (Figure 6a). Remarkably, after platinum adsorption, the CuFe_2_O_4_/HYPS sample showed an enhanced peak absorption up to 700 nm. A small absorption peak at about 224 nm showed the presence of tetrahedral coordinated Pt nanoclusters, while a significant enhancement of the absorption peak between 350 and 600 nm shows the presence of octahedral coordinated Pt species (Figure 6b). The presence of such strong bonds of tetrahedral and octahedral Pt species indicates the high dispersity and interaction of Pt on HYPS silica support.

**Figure 7:**
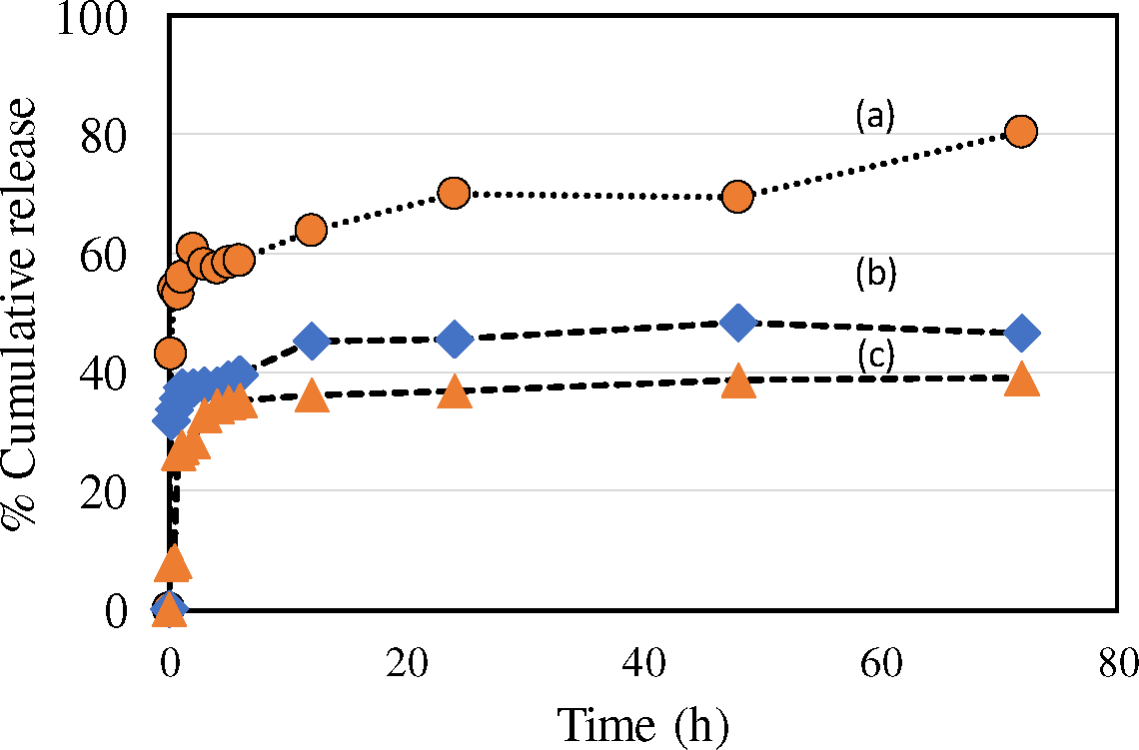
Percentage cumulative cisplatin release in tumor pH 5 conditions for 72 h in (a) CuFe_2_O_4_/HYPS, (b) CuFe_2_O_4_/AlMSU-F and (c) CuFe_2_O_4_/silicalite.

The drug release ability of CuFe_2_O_4_/HYPS was compared with two different supports, mesocellular foam and silicalite in simulated tumor acidic pH conditions (pH 5) at 37 °C for 72 h (Figure 7). The cisplatin (mmol) per gram of CuFe_2_O_4_/nanosupport was maintained at 0.15. In the case of cisplatin adsorption, three formulations showed adsorption between 86–90%. In particular, CuFe_2_O_4_/HYPS showed a high adsorption of about 88.6%. On the other hand, CuFe_2_O_4_/Al-MSU-F and CuFe_2_O_4_/silicalite showed an adsorption of up to 87.2% and 85.3%, respectively. Among the different nanoformulations, the order of cisplatin drug release was as follows: CuFe_2_O_4_/HYPS > CuFe_2_O_4_/Al-MSU-F > CuFe_2_O_4_/silicalite. CuFe_2_O_4_/HYPS showed the highest percentage cumulative cisplatin release of 80% over 72 h. The study shows the positive synergism of spherical silica HYPS with CuFe_2_O_4_ for cisplatin release under acidic tumor conditions. CuFe_2_O_4_/silicalite showed about 50% decreased cisplatin release compared to the HYPS support. The percentage release was about 46% and 39%, which indicates the positive effect of CuFe_2_O_4_/HYPS with respect to the cisplatin release rate. The trend signifies the importance of synergism between CuFe_2_O_4_, cisplatin and HYPS, which helps to release more cisplatin (Figure 7).

### In vitro anticancer studies

**Figure 8:**
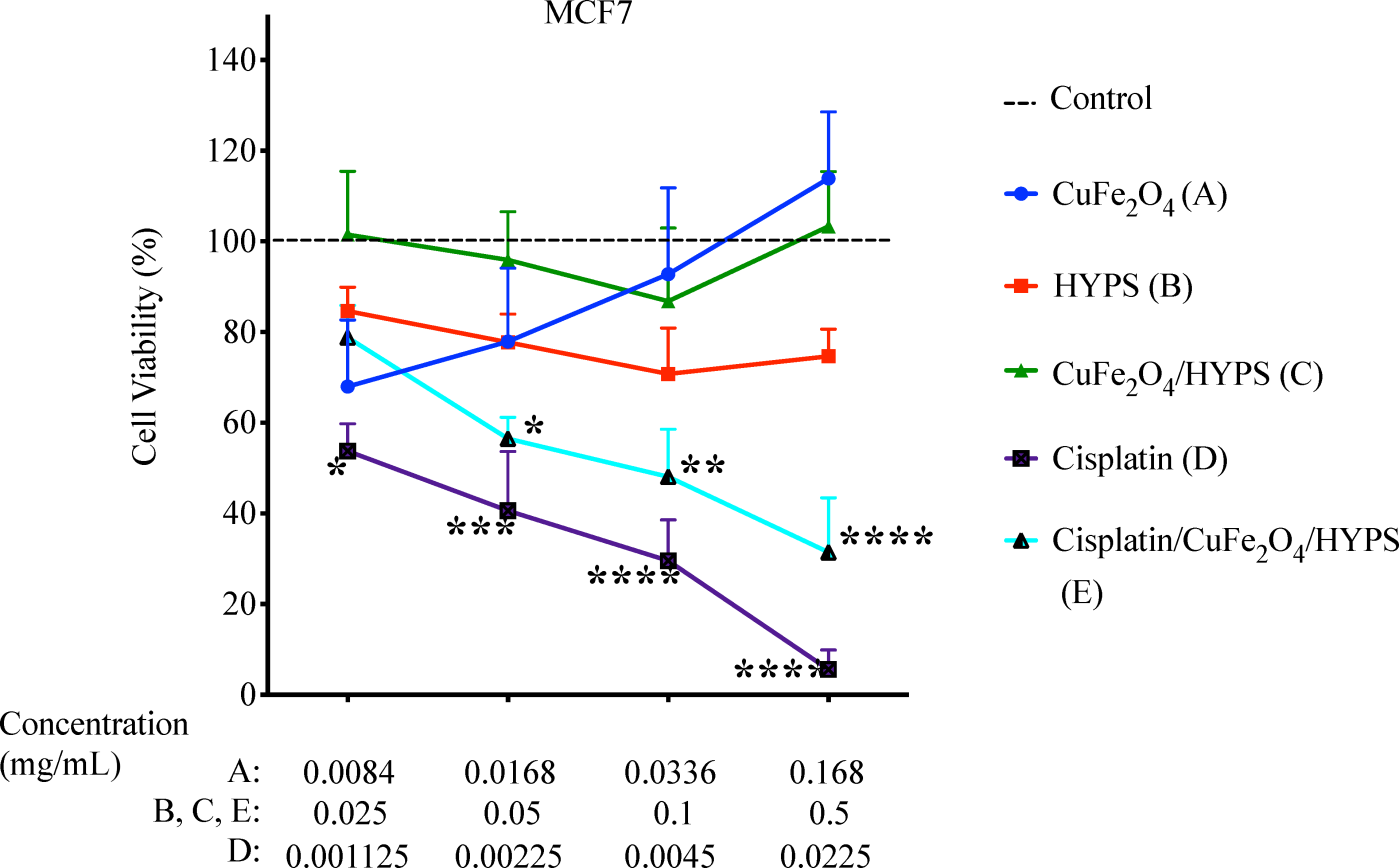
Percentage cell viability using MTT assay on the MCF-7 cell line. The cells were treated with the following formulations for 48 h: CuFe_2_O_4_ (A), HYPS (B), CuFe_2_O_4_/HYPS (C), cisplatin (D), and cisplatin/CuFe_2_O_4_/HYPS (E). The treatment concentrations used were as indicated in the figure. The reason for the different concentrations of CuFe_2_O_4_ and cisplatin is because they correspond to the amount adsorbed on the cisplatin/CuFe_2_O_4_/HYPS nanoparticles. For details, please see the Materials and Methods section. *n* = 4 independent experiments. Dashed line represents untreated control. Error bars ± standard error of the mean (SEM) and * *p* < 0.05; ** *p* < 0.01; *** *p* < 0.001; **** *p* < 0.0001 versus control.

**Figure 9:**
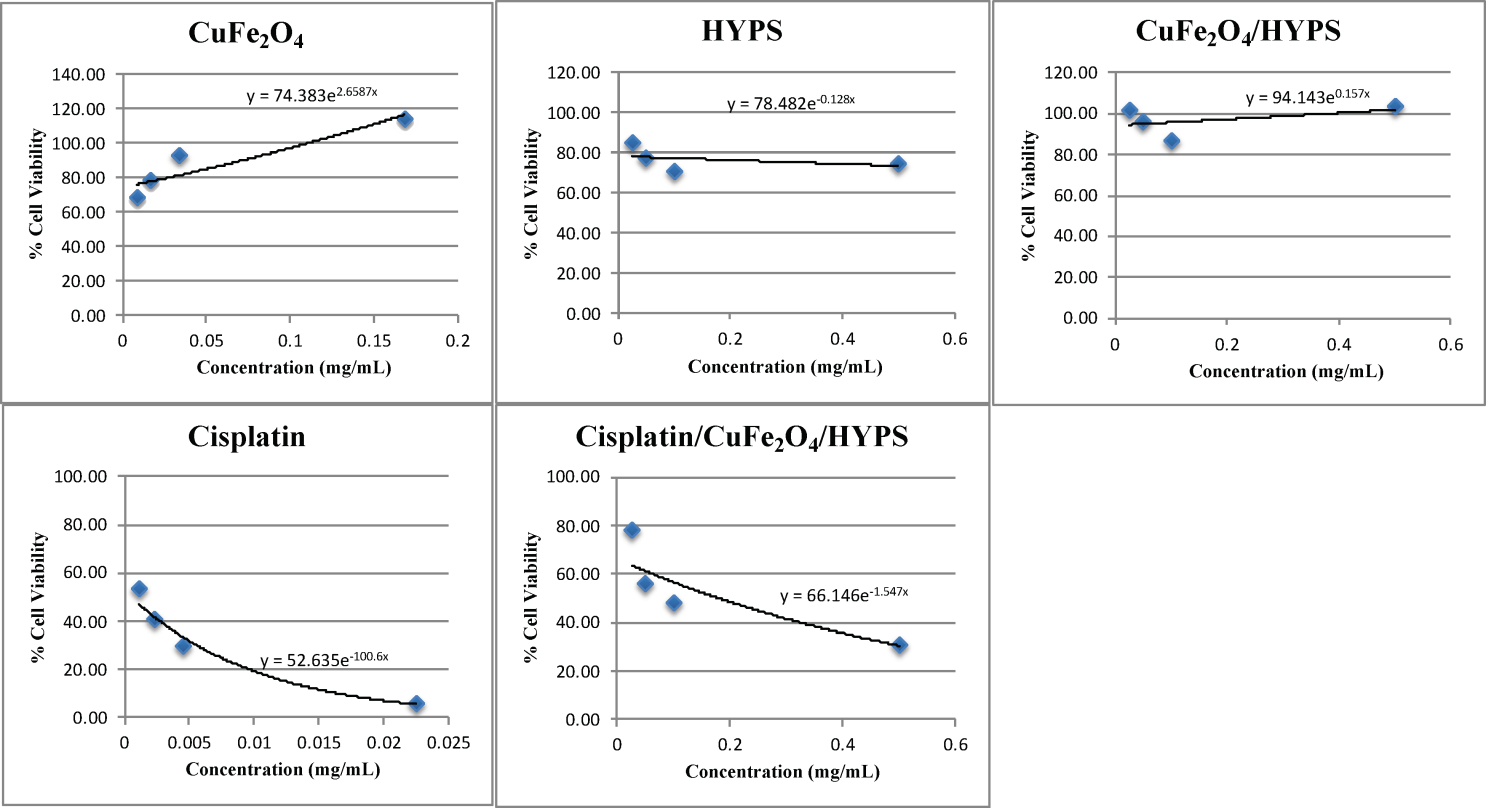
EC_50_ values for each condition. Data sets from Figure 8 were used to extrapolate the line equation of each condition: CuFe_2_O_4_, HYPS, CuFe_2_O_4_/HYPS, cisplatin, and cisplatin/CuFe_2_O_4_/HYPS.

Cisplatin is a well-known efficient anticancer drug that binds to DNA blocking cell division. As with many anticancer drugs, cisplatin has off-target toxicity, mainly in the kidneys, liver, heart, nerves, and ears. In addition, most patients develop chemoresistance to cisplatin [20–21]. To overcome these limitations and to ensure specific tumor targeting, cisplatin/CuFe_2_O_4_/HYPS nanoparticles were tested. To investigate the cytotoxic efficiency of cisplatin/CuFe_2_O_4_/HYPS nanoparticles, we assessed cell viability using the MTT assay. In that assay, healthy cells will be able to reduce MTT to the purple-colored formazan, while unhealthy/dead cells cannot. This test allows us to test the cytotoxic effects of cisplatin/CuFe_2_O_4_/HYPS on cancerous cells and its efficacy as a potential chemotherapeutic drug. Breast adenocarcinoma cell line, MCF7 cells were treated with the following conditions: CuFe_2_O_4_, HYPS, CuFe_2_O_4_/HYPS, cisplatin, and cisplatin/CuFe_2_O_4_/HYPS for 48 h (Figure 8, Figure 9 and Table 1). Cells that were treated with CuFe_2_O_4_, HYPS, and CuFe_2_O_4_/HYPS nanoparticles did not have a significant effect on cell viability. As expected, cells treated with the free cisplatin group (D) had a significant reduction in cell viability, which reached 53.8% at the lowest concentrations used. Cisplatin was able to maintain a steady reduction in cell viability as its concentration was increased. At the highest concentration (0.0225 mg/mL), free cisplatin resulted in 6% cell viability (Figure 8 and Table 1).

Interestingly, when cisplatin was loaded into CuFe_2_O_4_-coated silica nanoparticles (group E), it was still able to significantly reduce the cell viability in a dose dependent manner. At the highest concentration used (0.5 mg/mL), cisplatin/CuFe_2_O_4_/HYPS nanoparticles resulted in a 31.4% cell viability. Our results show that while CuFe_2_O_4_ and CuFe_2_O_4_/HYPS did not elicit a significant cytotoxic effect, cisplatin/CuFe_2_O_4_/HYPS nanoparticles were able to reduce the cellular viability (Figure 9 and Table 3). These results suggest that the cytotoxic effects observed on breast cancer cell line MCF7 were attributed to the cisplatin released from the cisplatin/CuFe_2_O_4_/HYPS nanoparticles.

**Table 3:** Significance and *p* values of experimental groups using the highest dose. * *p* < 0.05; ** *p* < 0.01; *** *p* < 0.001; **** *p* < 0.0001 versus control. N.S. indicates non-significant.

Drug group	Significance	*p* value

CuFe_2_O_4_	N.S.	0.8058
HYPS	N.S.	0.3059
CuFe_2_O_4_/HYPS	N.S.	0.9996
cisplatin	****	<0.0001
cisplatin/CuFe_2_O_4_/HYPS	****	<0.0001

The half-maximal effective concentration (EC_50_) is the concentration of a drug required to stimulate 50% of the response. It was calculated from the equation of the fitted line of cell viability values of each condition (Figure 9 and Table 4). The EC_50_ of CuFe_2_O_4_ and CuFe_2_O_4_/HYPS were negative values because cell viability did not decrease with increasing concentration (Figure 9). On the other hand, the EC_50_ of HYPS was 3.5 mg/mL, which means that for HYPS to induce a cytotoxic response it has to be administered at high concentrations. In contrast, the EC_50_ for cisplatin/CuFe_2_O_4_/HYPS nanoparticles was 0.18089 mg/mL and the free cisplatin was 0.00051 mg/mL. These results suggest that while free cisplatin was more potent than cisplatin/CuFe_2_O_4_/HYPS nanoparticles, the nanoparticles were still able to elicit a cytotoxic effect in MCF7 cells at low concentrations. Even though our results show a lower potency of cisplatin/CuFe_2_O_4_/HYPS nanoparticles than the classically administered cisplatin, the advantages of utilizing functionalized cisplatin in nanoparticles to prevent off-target effects, while allowing imaging and targeted delivery, outweighs these limitations. Specifically, the functionalized cisplatin is expected to prevent off-target effects, while the CuFe_2_O_4_ coating will allow targeted delivery of these nanoparticles.

**Table 4:** EC_50_ values of the various drug groups used in this in vitro study.

Drug group	Equation fit	EC_50_ value (mg/mL)

A	*y* = 74.383e^2.6587^*^x^*	−0.14940
B	*y* = 78.482e^−0.128^*^x^*	3.52224
C	*y* = 94.143e^0.157^*^x^*	−0.40305
D	*y* = 52.635e^−100.6^*^x^*	0.00051
E	*y* = 66.146e^−1.547^*^x^*	0.18089

Our results show that cisplatin/CuFe_2_O_4_/HYPS nanoparticles can effectively reduce the viability of the human breast cancer cell line MCF7. Thus, cisplatin/CuFe_2_O_4_/HYPS nanoparticles can be considered an interesting option for drug delivery (Scheme 1).

## Conclusion

An effective multifunctional CuFe_2_O_4_/HYPS nanocomposite system has been developed to deliver cisplatin. Samples containing 30 wt % CuFe_2_O_4_ loading showed the presence of cubic spinel consisting of tetrahedral and octahedral crystalline sites with a surface occupation of about 28% on the HYPS support. TEM and EDX mapping analysis showed the presence of homogeneous silica particles with nanoclusters (5–10 nm) of copper ferrite covering the HYPS. The composite showed paramagnetic behavior with a saturated magnetization value of 7.65 emu/g. The CuFe_2_O_4_/HYPS nanocomposite showed high cisplatin delivery and anticancer efficacy due to the coexistence of Pt and CuFe_2_O_4_ in HYPS. The cisplatin drug release ability using the HYPS support was found to be higher than when AlMSU-F and silicalite were used. The treatment of the breast cancer cell line MCF-7 with cisplatin/CuFe_2_O_4_/HYPS (Group E) showed a significant cytotoxic effect. It is also expected that the nanocomposite formulation will be able to circumvent the negative off-target effects that often accompany treatment with cisplatin, while at the same time, allowing for improved imaging and targeted drug delivery. Overall, the paramagnetic copper ferrite/HYPS nanocomposite developed in this work can be considered as a potential candidate for multifunctional theranostic applications, whereby the nanocomposite may be further engineered with biocompatible polymers, antioxidants and drugs.

## Supporting Information

File 1 Additional figure.
