# Supplementary material for: Targeted therapeutic effect against the breast cancer cell line MCF-7 with a CuFe2O4/silica/cisplatin nanocomposite formulation
Source: Beilstein J Nanotechnol. 2019 Nov 12;10:2217–28. doi: 10.3762/bjnano.10.214 (PMC6880833; doi:10.3762/bjnano.10.214)
Supplement: File 1 — Additional figure. [file Beilstein_J_Nanotechnol-10-2217-s001.pdf]

BEILSTEIN JOURNAL OF NANOTECHNOLOGY

# Supporting Information

for

# Targeted therapeutic effect against the breast cancer cell line MCF-7 with a $CuFe_2O_4$/silica/cisplatin nanocomposite formulation

B. Rabindran Jermy, Vijaya Ravinayagam, Widyan A. Alamoudi, Dana Almohazey, Hatim Dafalla, Lina Hussain Allehaibi, Abdulhadi Baykal, Muhammet S. Toprak and Thirunavukkarasu Somanathan

*Beilstein J. Nanotechnol.* **2019,** *10,* 2217–2228. doi:10.3762/bjnano.10.214

# Additional figure

License and Terms: This is a supporting information file under the terms of the Creative Commons Attribution License (http://creativecommons.org/licenses/by/4.0). Please note that the reuse, redistribution and reproduction in particular requires that the authors and source are credited.
The license is subject to the *Beilstein Journal of Nanotechnology* terms and conditions: (https://www.beilstein-journals.org/bjnano)

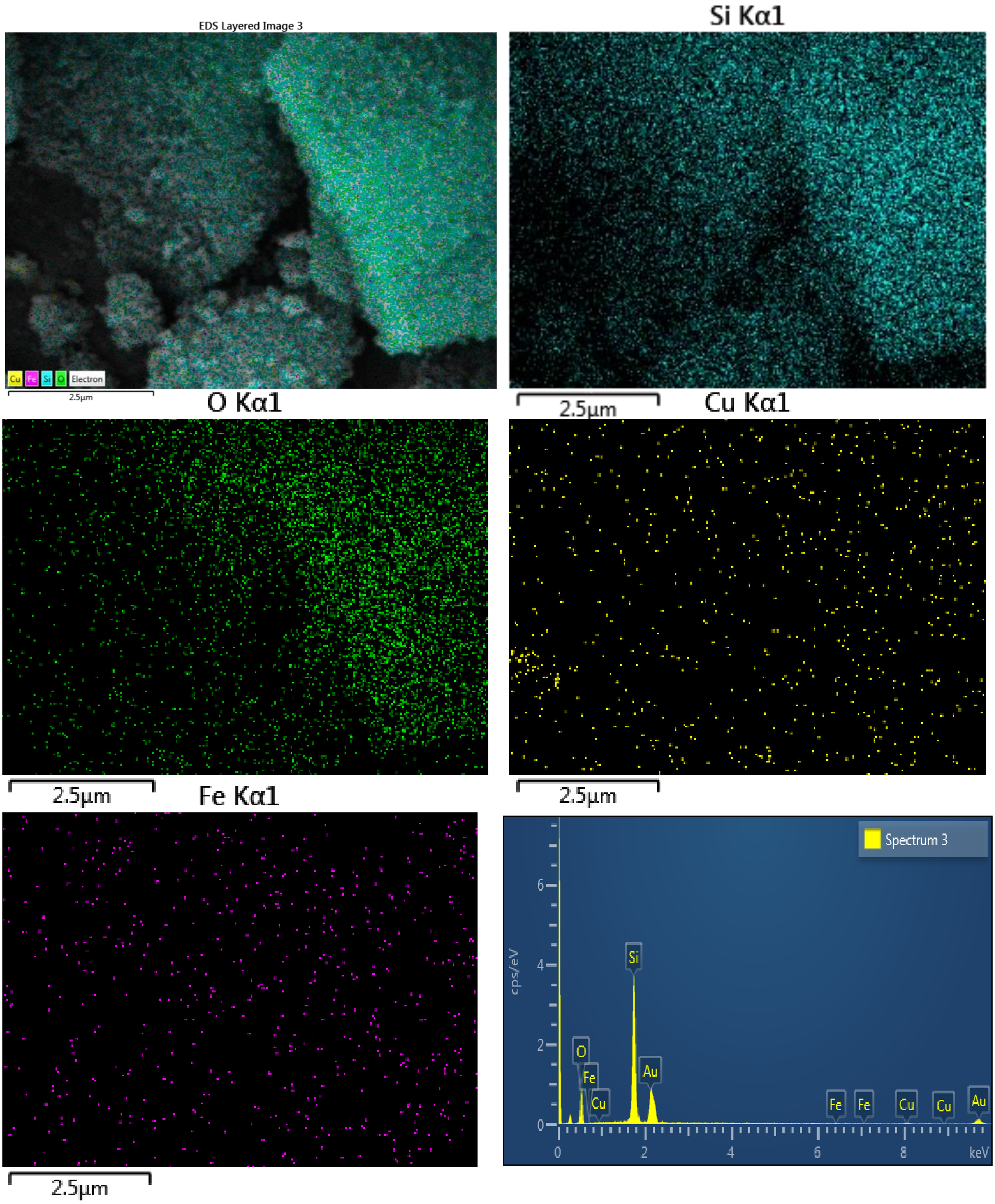


**Figure S1:** SEM-EDX analysis 30% $CuFe_2O_4$/HYPS.
